# A Case Report of a Successful Attempt to Create a Hemodialysis Vascular Access in a Patient With Recurrent Failed Arteriovenous Fistulas

**DOI:** 10.7759/cureus.47894

**Published:** 2023-10-29

**Authors:** Rajeshwar Yadav, Aditya Sharma, Swati Pathak

**Affiliations:** 1 Department of Cardiothoracic & Vascular Surgery, Institute of Medical Sciences, Banaras Hindu University, Varanasi, IND; 2 Department of General Surgery, Institute of Medical Sciences, Banaras Hindu University, Varanasi, IND

**Keywords:** radio-cephalic fistula, recurrent failed fistulas, difficult vascular access, arteriovenous fistula repair, chronic renal insufficiency

## Abstract

The majority of individuals undergoing hemodialysis for chronic renal insufficiency (CRI) require vascular access. The more appropriate and long-term accesses are arteriovenous fistulas (AVF). These accesses must be attempted to be salvaged even in the circumstances when they stop functioning.

In this study, a case report of a 57-year-old female patient with CRI who presented with a failed brachioradial and brachiocephalic AVF in the left upper limb and who later underwent the creation of a new functional radio-cephalic AVF mid-arm on the same limb is presented.

## Introduction

Creating an arteriovenous fistula (AVF) is a challenging task that provides sustainable vascular access (VA) for hemodialysis (HD) [1]. There is general agreement that an arteriovenous fistula (AVF) with an autogenous vein is the ideal vascular access for these patients [2]. Numerous studies highlight the significance of AVF preservation and treatment in extending its "useful life" span [3]. Thrombosis, stenosis, pseudoaneurysms, and infection are the most frequent vascular access-related challenges. Even in the face of these occurrences, it is sometimes feasible to make attempts to maintain access [4]. Techniques like thrombectomy to treat early occlusions, vein interposition for the treatment of pseudoaneurysms, endovascular procedures for stenosis, and the creation of new AVFs are other modalities to treat recurrent failed AVFs [5]. A review of the literature reveals the fact that it may be possible to salvage AVFs in patients with long-term failure.

## Case presentation

A 57-year-old female patient who had been receiving hemodialysis for 12 years presented with a failed brachioradial and brachiocephalic AVF. The patient denied having ever used a central venous catheter. She experienced pain in her left upper limb during hemodialysis that had subsided over the previous month, along with the AVF bruit.

**Figure 1:**
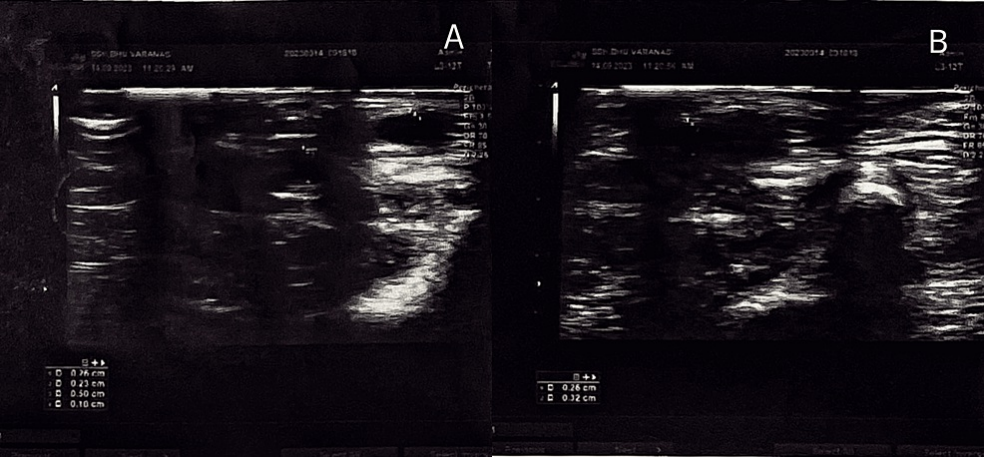
A bilateral upper limb color Doppler film showing blood flow in the (A) right upper limb and (B) left upper limb

Physical examination revealed no bruit or thrill along the cephalic vein's entire course until a brief venous segment (approximately 5 cm) at the anterior part of the distal arm showed a 3+/4+ bruit or thrill. The basilic vein, which runs from the elbow to the proximal portion of the left arm, was also rumbling or throbbing. There was no edema or collateral venous circulation. A color Doppler bilateral upper limb for AVF was advised and was interpreted as shown in Figure 1 and elaborated in Table 1.

**Table 1:** A table showing color Doppler findings and vessel diameter in both upper limbs in cm (centimeter)

Region of the upper limb	Right	Left
Cubital fossa		
Brachial artery diameter	0.27 cm	0.36 cm
Cephalic vein diameter	0.22 cm	0.17 cm
Skin distance of the brachial artery	0.35 cm	0.20 cm
Skin distance of the cephalic vein	0.13 cm	0.13 cm
Distance between the brachial artery and cephalic vein	1.07 cm	1.91 cm
Mid arm		
Radial artery diameter	0.15 cm	0.19 cm
Cephalic vein diameter	0.14 cm	0.24 cm
Skin distance of the radial artery	0.43 cm	0.39 cm
Skin distance of the cephalic vein	0.09 cm	0.18 cm
Distance between the radial artery and cephalic vein	0.80 cm	1.32 cm
Wrist		
Radial artery diameter	0.17 cm	0.23 cm
Cephalic vein diameter	0.16 cm	0.17 cm
Skin distance of the radial artery	0.25 cm	0.29 cm
Skin distance of the cephalic vein	0.09 cm	0.09 cm
Distance between the radial artery and cephalic vein	0.55 cm	0.99 cm

**Figure 2:**
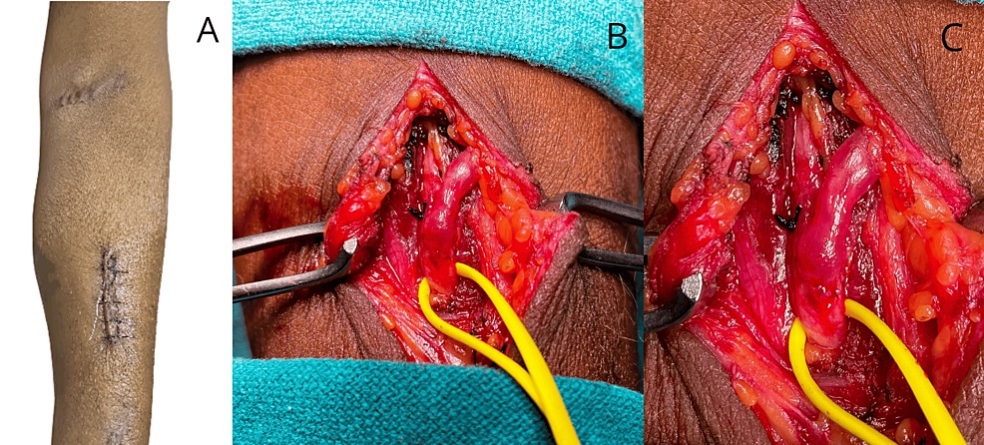
(A) A picture showing two failed fistulas in the left forearm; (B) an intraoperative picture showing a cephalic vein right after dissection; (C) a cephalic vein placed on the suprafascial level after the confection of the subcutaneous level

Based on the color Doppler findings, a decision was made to create an AVF in between radiocephalic vessels, and consequently, a new fistula in between the two failed brachioradial and brachiocephalic AVFs was created, as shown in Figure 2.

The patient was discharged on postoperative day two following an uneventful postoperative period. Subsequently, the fistula matured after six weeks of creation and was fully functional.

## Discussion

A common therapy option for individuals with end-stage renal failure is hemodialysis. Compared to prosthetic grafts or dialysis catheters, arteriovenous fistula creation is associated with a longer maturation period but a lower incidence of infection, hospitalizations, central venous stenosis, and mortality [6]. The maturation of dialysis fistulas is a dynamic process known as venous arterialization, in which the release of nitrous oxide and the breakdown of elastin enable the structural and functional remodeling of the venous wall, allowing the output vein to expand [7].

Clinically matured arteriovenous fistulas offer the high flow rates required to maintain hemodialysis and are simpler to cannulate with repeated needle sticks. The arteriovenous fistula should, six weeks after creation, reach the following criteria: a blood flow rate of at least 600 ml/min, a diameter of at least 6 mm, a length of at least 6 cm for cannulation access, and a depth of 6 mm or less from the skin surface. This is known as the "Rule of 6's" and is used by the Kidney Disease Outcomes Quality Initiative (KDOQI) to evaluate fistula maturation [8].

A successful hemodialysis access fistula is one that is durable, possesses a low risk of infection, and requires only minor upkeep for continuous functioning. It is widely acknowledged that mature AVF outperforms prosthetic arteriovenous grafts in terms of overall patency, revision rate, and cost savings [9].

Duplex ultrasonography scanning and venography during preoperative evaluation may help identify veins that are not observable during a clinical examination, allowing for the placement of functional autogenous fistulas with an extended lifespan [10]. The success of the procedure prevented the necessity for a central venous catheter and all of its difficulties, enhanced the patient's hemodialysis experience, and accomplished so without the need for any extra venous segments or prostheses. This will enable the earliest possible beginning of hemodialysis and minimize the morbidity linked to additional surgical procedures.

## Conclusions

The effectiveness of dialysis fistulas in patients with end-stage renal illness depends on an interdisciplinary team approach that includes primary care physicians, nephrologists, vascular access surgeons, interventionalists, dialysis nurses, and vascular access coordinators working together. In this particular case, vascular condition and patency were crucial clinical predictors that could direct the selection of the AVF development site, assisting in the effective establishment of the AVF.
